# Temporal Muscle Thickness Is a Prognostic Factor for Neurological Recovery After Surgery for Chronic Subdural Hematoma

**DOI:** 10.3390/diagnostics16091279

**Published:** 2026-04-24

**Authors:** Nikolina Šilješ, Zara Miočić, Irina Bagić, Zdravka Krivdić Dupan, Dario Mužević, Marina Vekić Mužević, Bruno Splavski, Barbara Šimatić, Karla Šutalo, Anja Radin Major, Nenad Nešković

**Affiliations:** 1 Department of Radiology, University Hospital Osijek, 31000 Osijek, Croatia; nikolina.grbavac91@gmail.com (N.Š.); zdravka.krivdic@gmail.com (Z.K.D.); 2 Department of Neurosurgery, University Hospital Centre Zagreb, 10000 Zagreb, Croatia; miociczara@gmail.com; 3 Department of Neurosurgery, University Hospital Osijek, 31000 Osijek, Croatia; irina.bagic@gmail.com (I.B.); dario.muzevic@me.com (D.M.); 4 Faculty of Medicine, Josip Juraj Strossmayer University of Osijek, 31000 Osijek, Croatia; marina.vekic.muzevic@me.com; 5 Department of Dermatology, University Hospital Osijek, 31000 Osijek, Croatia; 6 University of Applied Health Sciences, 10000 Zagreb, Croatia; splavuno@gmail.com; 7 Department of Surgery Service of Neurosurgery, Dubrovnik General Hospital, 20000 Dubrovnik, Croatia; 8 Health Centre Osijek-Baranja County, 31000 Osijek, Croatia; pejcic.barbara@gmail.com; 9 Health Centre Zagreb, 10000 Zagreb, Croatia; sutalokarla7@gmail.com; 10 Department of Clinical Transfusion, Osijek University Hospital Osijek, 31000 Osijek, Croatia; anjaradin@gmail.com; 11 International Medical Center Priora, 31431 Čepin, Croatia

**Keywords:** chronic subdural hematoma, sarcopenia, temporal muscle thickness, outcome

## Abstract

**Background**: Sarcopenia is increasingly recognized as a prognostic factor in surgical populations. This study evaluated the association between cranial CT-based markers of sarcopenia and neurological outcomes in patients undergoing surgery for chronic subdural hematoma (CSDH). **Methods**: This retrospective case–control study included 82 patients who underwent surgery for unilateral CSDH. Demographic data, comorbidities, use of anticoagulant and antiplatelet therapy, postoperative complications and length of hospital stay were collected from patients’ medical records. Radiological parameters of sarcopenia, including temporal muscle thickness, temporal muscle area, and occipital fat pad thickness, as well as standard radiological features of CSDH, were measured preoperatively on the initial CT scan. Neurological outcome 3 months after surgery was assessed using the Glasgow Outcome Scale, with scores ≥ 4 defined as favourable and scores 1–3 as poor. **Results**: Demographic and clinical characteristics, including age, sex, comorbidities, hematoma thickness and intracranial midline shift, did not differ significantly between outcome groups. Temporal muscle thickness (4.7 vs. 2.8 mm, *p* < 0.001), temporal muscle area (160 vs. 106 mm^2^, *p* = 0.04), and occipital fat pad thickness (4.7 vs. 3.4 mm, *p* = 0.04) were significantly greater in patients with favourable neurological outcomes. After corrections for age and comorbidities, multivariate logistic regression with temporal muscle thickness, area and density, temporal bone thickness and density, and occipital fat pad thickness demonstrated that temporal muscle thickness was the only independent predictor of good neurological recovery (OR 3.20, 95% CI 1.37–7.46, *p* = 0.007). ROC analysis showed good discriminatory power of temporal muscle thickness (AUC 0.812, 95% CI 0.695–0.930, *p* < 0.001), with a cut-off value of ≥3.37 mm for its ability to predict favourable neurological outcome. **Conclusions**: Temporal muscle thickness is a reliable, non-invasive imaging biomarker for predicting good neurological recovery after CSDH surgery and may aid in risk stratification, particularly in elderly or frail patients.

## 1. Introduction

Chronic subdural hematoma (CSDH) is a prevalent neurosurgical condition, particularly in older adults, and is often associated with significant morbidity and mortality [1]. Despite the widespread use of surgical interventions, predicting neurological outcomes in CSDH patients remains challenging due to the complex pathophysiology of the condition. Complementary to improving treatment strategies, identifying particularly vulnerable CSDH patients would allow interventions to be targeted toward those at highest risk [2]. Sarcopenia, characterized by the progressive loss of skeletal muscle mass and function, has emerged as a significant predictor of poor health outcomes in the ageing population. Recent studies have highlighted its association with increased risk of frailty, disability, and mortality in various conditions, such as stroke, aneurysmal subarachnoid hemorrhage, glioblastoma, head and neck carcinoma, chronic subdural hemorrhage and traumatic brain injury [3,4,5,6,7,8]. Radiological markers, particularly those identified on computed tomography (CT) scans or magnetic resonance imaging, offer a non-invasive means of assessing sarcopenia. These markers include measurements of muscle thickness, cross-sectional area, and muscle density, all of which can be quantified through standard imaging techniques [9]. The objective of this study was to evaluate the influence of radiological markers of sarcopenia on neurological outcomes in patients with CSDH, aiming to assess their potential utility as prognostic indicators in clinical settings.

## 2. Materials

Upon obtaining approval from the Ethics Committee, a retrospective analysis was conducted on the medical records of patients admitted to the University Hospital Osijek due to CSDH. The study included patients over 18 years of age who were surgically treated for unilateral CSDH between November 2018 and October 2023. Patients with bilateral hematomas or additional intracranial pathology were excluded from the analysis. Patients included in the study underwent a surgical evacuation of subdural hematoma with the use of body temperature saline irrigation, followed by subdural drainage placement. Demographic data, comorbidities, anticoagulant and antiplatelet therapy, postoperative complications (including local and systemic infections, hemorrhage, hematoma recurrence, epileptic seizures and thromboembolism), length of hospital stay, and treatment outcome assessed by Glasgow Outcome Scale (GOS) were collected from patients’ medical histories. The neurological outcome was categorized as favourable (GOS 4 and 5) or poor (GOS 1–3) [10]. Additionally, CT image analysis was used to determine the thickness of the CSDH, midline shift, and the thickness, density, and area of the left and right temporal muscles (TMs), the thickness and density of the left and right temporal bones (TBs), and the thickness of the occipital fat pad (OF). For measurements taken bilaterally, the average value of the left and right sides was used in the analysis.

### 2.1. Measurement of the Temporal Muscle, Temporal Bone Density, Occipital Fat Pad, Midline Shift and CSDH

**Figure 1:**
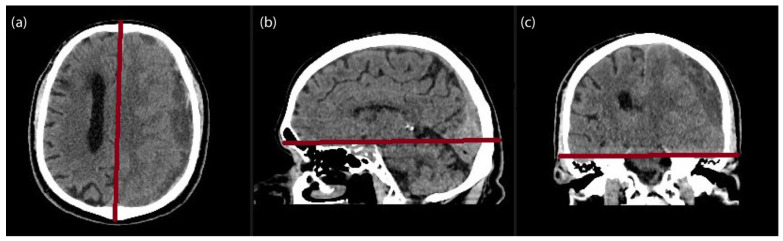
The axial (**a**), coronal (**b**) and sagittal (**c**) localisation planes (red lines) used to align the scan to produce the semi-axial plane.

Preoperative CT were performed using a multisectioned CT scanner (Somarom X.cite; Siemens, Munich, Germany). The measured temporal muscle thickness (TMT), temporal muscle area (TMA), temporal muscle density (TMD), temporal bone thickness (TBT), temporal bone density (TBD), occipital fat pad (OF), midline shift and CSDH thickness were evaluated manually by a radiologist using the Picture Archiving and Communication System (PACS) imaging software (Sectra IDS7, Version 24.1, Linkoping, Sweden), blinded to the patient’s outcomes. Multiplanar reconstructions were generated along standardized neuroanatomical planes. The semi-axial plane was aligned parallel to the anterior skull base, the sagittal plane was oriented parallel to the falx cerebri, and the coronal plane was positioned tangential to the floor of the middle cranial fossae (Figure 1).

**Figure 2:**
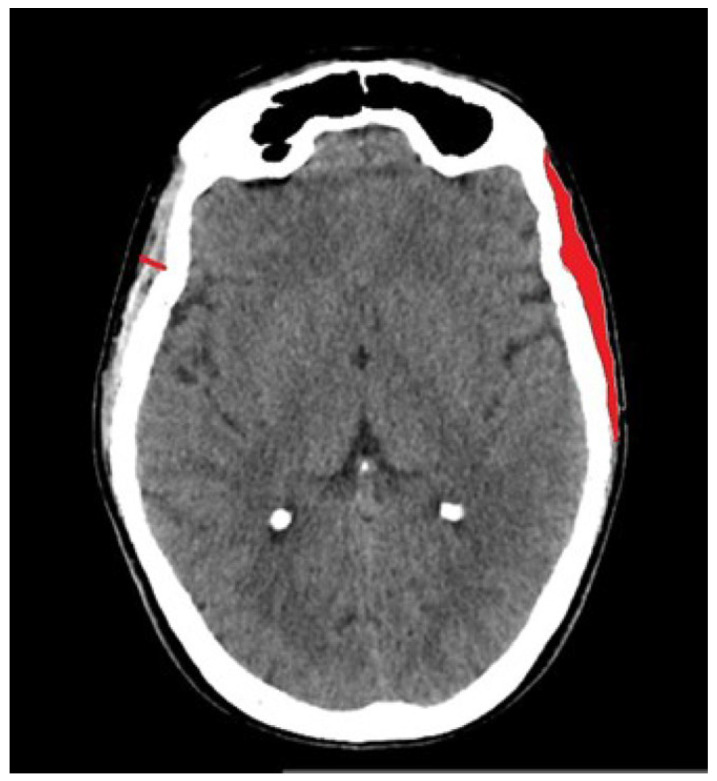
Representative case for the assessment of the temporal muscle thickness (red line) and temporal muscle area (red area).

The TMT was measured on the first axial slice above the bony orbit. All the other measurements were taken on the same slice. It was measured perpendicular to the long axis of the muscle, typically at its thickest portion on both the left and right sides. Measurements were taken from the outer border of the temporal fascia to the inner border adjacent to the skull. Values from both sides are average. The TMA was evaluated manually by tracing the outline of the temporal muscle, and values from both sides were averaged (Figure 2). The TMD was measured by placing the region of interest (ROI) in the traced area. The TBT was measured on the level of Sylvian fissure perpendicularly from the outer to the inner cortical surface of the temporal bone. The TBD was measured at the same point by manually placing round or elliptical ROIs. Midline shift was measured as the longest perpendicular distance of the septum pellucidum from a sagittal line connecting the anterior and posterior bony insertions of the falx cerebri. CSDH was measured on an axial slice perpendicular to the anterior–posterior axis of the head with maximum thickness of the hematoma. Maximal perpendicular distance was measured between the inner table of the skull. For the OF linear measurement, the maximum thickness of the fat pad was measured in the midline, perpendicularly from the outer surface of the occipital bone to the skin surface.

### 2.2. Statistical Analysis

Categorical data are presented as absolute and relative frequencies, while numerical data are presented as medians and interquartile ranges. The normality of the distribution of numerical variables was tested using the Shapiro–Wilk test. Comparisons between two groups were made using Fisher’s exact test for categorical variables and the Mann–Whitney U test for continuous variables. The correlation between radiographic indicators of sarcopenia and neurological outcomes was assessed using Spearman’s rank correlation test. To evaluate the association between independent variables and treatment outcomes, univariate and multivariate logistic regression was conducted. Receiver operating characteristic (ROC) curve analysis, including the calculation of 95% confidence intervals (CIs), was used to assess the predictive accuracy of sarcopenia indicators for neurological outcomes. The optimal cut-off values were identified using Youden’s index. Statistical significance was defined as a two-tailed *p*-value of less than 0.05. Analyses were performed using IBM SPSS Statistics for Macintosh, Version 28.0 (IBM Corp., Armonk, NY, USA).

## 3. Results

### 3.1. Patient Characteristics and Neurological Outcome

A total of 82 patients who underwent surgery for CSDH were analyzed. A total of 71 patients (86.6%) had a good neurological outcome (GOS 4–5), while 11 patients (13.4%) had a poor outcome (GOS 1–3). There were no significant differences between the two groups in terms of age, sex, comorbidities, or the use of antiplatelet or anticoagulant therapy. Similarly, hematoma thickness and midline shift on radiographic imaging did not differ significantly between outcome groups. However, patients with poor neurological outcomes were significantly more likely to experience postoperative local infection or subsequent local hemorrhage (Table 1).

### 3.2. Radiological Markers of Sarcopenia and Neurological Outcome

A significant positive correlation was observed between a favourable neurological outcome and TMT (Spearman’s rho = 0.41, *p* < 0.001), TMA (Spearmen’s rho = 0.25, *p* = 0.02), and OF thickness (Spearman’s rho = 0.23, *p* = 0.03). Other radiological markers of sarcopenia did not show a significant correlation with neurological outcome.

TMT and TMA, as well as OF thickness, were significantly greater in patients with a favourable neurological outcome (Table 2).

Results of the univariate logistic regression analysis show that TMT is the only significant predictor of favourable neurological outcome (OR 2.22, 95% CI 1.26–3.90, *p* = 0.006). Other variables did not reach statistical significance, although the occipital fat pad was borderline significant and showed a trend toward a favourable outcome (OR 1.35, 95% CI 0.99–1.83, *p* = 0.05) (Table 3).

After corrections for age and comorbidities (hypertension, diabetes, alcoholism, antiaggregating and anticoagulation therapy), multivariate regression analysis—including radiological markers of sarcopenia—identified TMT as the only significant predictor of a good neurological outcome (OR 3.20, 95% CI 1.37–7.46, *p* = 0.007). The multivariate model was statistically significant overall (χ^2^ (7) = 19.532, *p* = 0.007) and correctly classified 89% of cases (Table 4).

**Figure 3:**
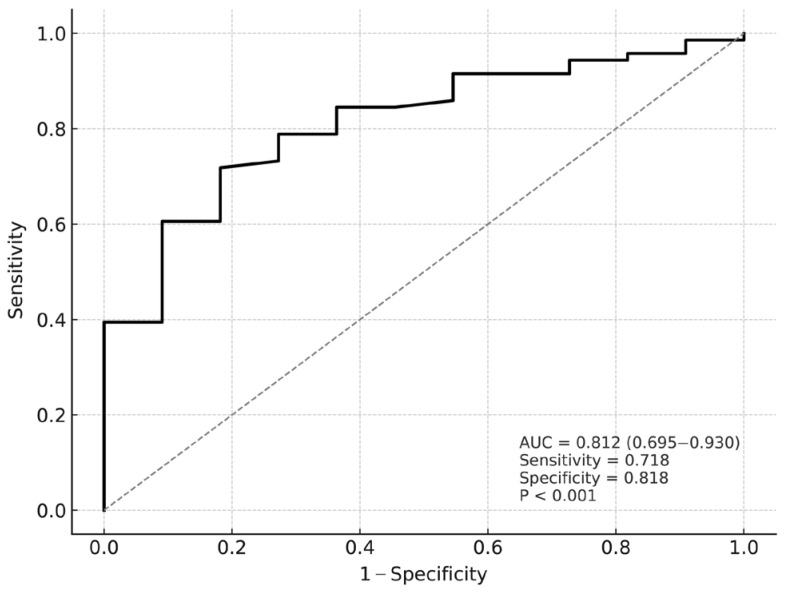
ROC curve for temporal muscle thickness predicting neurological outcome in chronic subdural hematoma. AUC: area under the curve.

TMT demonstrated significant prognostic value for neurological outcome, as shown by ROC curve analysis (AUC = 0.812; 95% CI: 0.695–0.930; *p* < 0.001). A cut-off value of ≥3.37 mm predicted a favourable neurological outcome with a sensitivity of 71.8% and a specificity of 81.8% (Figure 3).

Further subgroup analysis by sex revealed that TMT maintained its prognostic significance in both female and male patients. In female patients, ROC curve analysis yielded an AUC of 0.824 (95% CI: 0.646–1.000, *p* < 0.001), with an optimal cut-off value of ≥2.9 mm, resulting in a sensitivity of 76.5% and a specificity of 100%. In male patients, the AUC was 0.810 (95% CI: 0.670–0.950, *p* < 0.001) with a cut-off value of ≥3.8 mm, achieving a sensitivity of 64.8% and a specificity of 87.5% in predicting favourable outcomes.

## 4. Discussion

The results of this study showed that TMT, TMA, and OF on preoperative CT scans were significantly associated with favourable neurological outcomes, with TMT emerging as the most robust and independent predictor. Sarcopenia measured at the level of the third lumbar vertebra (L3) is a well-established imaging biomarker associated with adverse outcomes in oncological and surgical populations [11,12]. As abdominal imaging is not always available, recent research has explored alternative markers, with TMT emerging as a viable surrogate due to its correlation with L3-based measurements [13,14]. These findings support growing evidence that cranial imaging-derived muscle metrics, particularly TMT, can serve as practical, non-invasive biomarkers of patient frailty and prognosis in the neurosurgical population [7,12,15,16]. Current evidence suggests that reduced muscle thickness may reflect a broader state of physiological vulnerability, including frailty, impaired metabolic reserve, chronic inflammation, and reduced capacity to respond to surgical stress [17,18,19]. In this context, TMT may serve as an imaging-based surrogate marker of overall physiological reserve rather than a direct causal factor, with the additional advantage of being readily obtainable from routine imaging without imposing any additional burden on the patient, which is particularly valuable in acute neurosurgical settings where comprehensive frailty assessments are often not feasible. To the best of our knowledge, there are currently no published studies on OF as a prognostic marker, but this study suggests it may reflect overall subcutaneous fat reserves and systemic physiological status as an important prognostic factor. Although less predictive than TMT, OF could still hold value in assessing recovery potential, especially when standard body composition measures are unavailable. Further validation is needed [20]. In prior work on TMT in patients with CSDH, Dubinski et al. reported that TMT was associated with a higher degree of disability at discharge and at 3 months [8]. Notably, this association did not remain significant in their multivariate model in contrast with our results. Interestingly, while TMA and OF thickness were significantly higher in patients with favourable outcomes, they did not retain significance in multivariate models. This may be due to measurement variability, body composition differences, or the limited sample size. Also, we measured TMA on a single slice, whereas Katsuki et al. used measurements from three consecutive slices, which may offer greater reproducibility and a more accurate reflection of true muscle mass than a single cross-sectional area [21]. Additionally, it is important to highlight that the non-significant relationship between age and outcome in our multivariate model suggests that sarcopenia-related markers like TMT may be better indicators of functional capacity than chronological age alone [22,23]. Indeed, muscle mass appears to reflect biological rather than chronological age, possibly explaining its prognostic effect across all age groups. This builds on the body of evidence associating reduced muscle mass with sarcopenia, frailty, and malnutrition, all of which are geriatric syndromes and contribute to poor patient outcomes [24,25,26]. As treatment options for CSDH evolve—including minimally invasive approaches like middle meningeal artery embolization—there is a growing need for objective tools to guide treatment selection, especially in frail patients [27,28]. TMT may serve as such a tool, helping clinicians assess vulnerability and personalize care. Future research should explore TMT’s prognostic value in minimally invasive CSDH treatment, where accurate assessment of recovery potential is crucial, particularly in older patients who might otherwise be excluded from invasive treatment based solely on age. At present, TMT should not be interpreted as a standalone criterion for surgical decision-making. TMT may serve as an additional parameter contributing to risk stratification and perioperative planning—patients identified as having low TMT may potentially benefit from closer perioperative monitoring and optimized supportive care; however, specific therapeutic strategies remain to be clearly defined.

This study has several limitations that should be acknowledged. First, its retrospective design may introduce selection bias and limits the ability to establish causality. Additionally, all patients included were treated at a single tertiary care centre, which may restrict the generalizability of the findings to broader or more diverse populations. Variations in surgical techniques, postoperative management, and demographic characteristics across other institutions could influence clinical outcomes and thus may not be reflected in this cohort. Furthermore, the inflammatory status, an important factor known to impact both sarcopenia and neurological recovery, was not assessed due to lack of available data. The inclusion of inflammatory biomarkers such as C-reactive protein, interleukin-6, or the neutrophil-to-lymphocyte ratio could have provided valuable additional insight.

## 5. Conclusions

In patients undergoing surgery for chronic subdural hematoma, greater temporal muscle thickness, as measured by routine preoperative imaging, was significantly associated with favourable neurological outcomes. This supports the potential utility of TMT as a non-invasive biomarker for identifying patients with greater physiological reserve and better recovery potential. Future prospective studies incorporating comprehensive assessments of nutritional and inflammatory status are warranted to further validate the prognostic value of TMT in this context.

## Figures and Tables

**Table 1:** Patient demographics, comorbidities, and postoperative complications according to neurological outcome.

	Median (IQR) or n (%)		
	Favourable Neurologic Outcome (N = 71)	Poor Neurologic Outcome (N = 11)	*p* *
Age (years)	76 (67–83)	80 (76–82)	0.17
Sex (male)	54 (76.1)	8 (72.2)	>0.99
Comorbidities			
Hypertension	48 (67.6)	9 (81.8)	0.49
Diabetes mellitus	13 (18.3)	2 (18.2)	>0.99
Anticoagulation therapy	9 (12.7)	2 (18.2)	0.64
Antiaggregating therapy	13 (18.3)	1 (9.1)	0.68
Alcoholism	5 (7)	1 (9.1)	>0.99
Hematoma thickness (mm)	19.7 (11.3–25.9)	17.1 (14.2–22.3)	0.39
Midline shift (mm)	6.7 (4.1–11.2)	6.3 (0–9.1)	0.13
Postoperative complications			
Local infection	1 (1.4)	2 (18.2)	0.04
Hemorrhage	1 (1.4)	2 (18.2)	0.04
Epilepsy	6 (8.5)	2 (18.2)	0.29
Pneumonia	5 (7)	1 (9.1)	>0.99
Urinary tract infection	10 (14.1)	1 (9.1)	>0.99
Thromboembolism	1 (1.4)	0 (0)	>0.99
Length of hospital stay (days)	7 (5–10)	9 (6–16)	0.23

* Mann–Whitney test for continuous and Fisher’s exact test for categorical data.

**Table 2:** Radiological characteristics of sarcopenia markers according to neurological outcome.

	Median (IQR) or n (%)		
	Favourable Neurologic Outcome (N = 71)	Poor Neurologic Outcome (N = 11)	*p* *
TM thickness (mm)	4.7 (3.1–6.1)	2.8 (1.9–3.3)	<0.001
TM area (mm^2^)	160 (98–221)	106 (86–134)	0.04
TM density (HU)	48 (39–52)	44 (31–56)	0.79
TB thickness (mm)	6.5 (5.6–7.1)	6.4 (4.9–7.2)	0.59
TB density (HU)	743 (656–880)	647 (567–790)	0.18
Occipital fat pad (mm)	4.7 (3.5–6.7)	3.4 (1.2–5.5)	0.04

* Mann–Whitney test; TM: temporal muscle; TB: temporal bone; HU: Hounsfield unit.

**Table 3:** Univariate logistic regression analysis for predictors of favourable neurological outcome.

Independent Variables	β	Wald	*p*	Odds Ratio (95% CI)
TM thickness (mm)	0.797	7.677	0.006	2.22 (1.26–3.90)
TM area (mm^2^)	0.011	3.644	0.06	1.01 (1.00–1.02)
TM density (HU)	0.009	0.115	0.73	1.01 (0.96–1.06)
TB thickness (mm)	0.078	0.165	0.68	1.08 (0.74–1.57)
TB density (HU)	0.003	2.548	0.11	1.00 (0.99–1.01)
Occipital fat pad (mm)	0.300	3.695	0.05	1.35 (0.99–1.83)

CI: confidence interval; TM: temporal muscle; TB: temporal bone; HU: Hounsfield unit.

**Table 4:** Multivariate logistic regression analysis for predictors of favourable neurological outcome.

Independent Variables	β	Wald	*p* *	Odds Ratio (95% CI)
TM thickness (mm)	1.162	7.230	0.007	3.20 (1.37–7.46)
Constant	5.323	1.374	0.24	

* Corrected for age and comorbidities; CI: confidence interval; TM: temporal muscle.

## Data Availability

The data presented in this study are available on request from the corresponding author.
